# The role of the environment: how mask wearing varies across different activities

**DOI:** 10.1186/s12889-024-18142-4

**Published:** 2024-06-10

**Authors:** Ciara Nestor, Giulia Earle-Richardson, Christine E Prue

**Affiliations:** Social, Behavioral, and Evaluation Sciences Team, Office of the Director, National Center for Emerging and Zoonotic Infectious Diseases, Centers for Disease Control and Prevention, Atlanta, GA USA

**Keywords:** COVID-19, Face mask wearing, Latent class analysis

## Abstract

**Background:**

People’s decisions to engage in protective health behaviors, such as mask wearing during the COVID-19 pandemic, are influenced by environmental and social contexts. Previous research on mask wearing used a single question about general mask usage in public, which may not reflect actual behavior in every setting. The likelihood of wearing a mask during one activity is also related to the likelihood of wearing a mask in another or avoiding an activity entirely. This analysis compared responses between a general question and activity-specific questions and identified patterns of mask-wearing behavior across activities.

**Methods:**

Online, opt-in, cross-sectional surveys were conducted every 2 months from November 2020 to May 2021 (*n* = 2508), with quota sampling and weighting to achieve a representative sample of the U.S.population. Respondents were asked how frequently they wore a mask in public and during 12 specific activities including: on public transportation, while shopping, and attending social gatherings indoors and outdoors. Spearman’s rank order correlation was used to compare the frequency of mask wearing reported using a general question versus an activity specific question. Additionally, a latent class analysis was conducted to identify patterns of mask wearing behavior across activities.

**Results:**

There was little to no correlation (*r* = .16–0.33) between respondents’ general attitudes towards mask wearing and their reported frequency of mask wearing in different activities. Latent class analysis identified six distinct groups based on their mask wearing behaviors and avoidance of certain activities. The largest group (29%) avoided ten of the twelve activities and always wore a mask during activities that could not be avoided. Additional groups included those who avoided most activities but made exceptions when around friends or family (20%), part time mask wearers (18%), and never mask wearers (6%).

**Conclusions:**

The findings suggest that activity-specific questions provide more accurate and useful information than a single general question. Specific, context based, questions allow for analyses that consider the nuances of people’s decision-making regarding engaging in protective health behaviors, such as mask wearing, thus enabling public health professionals to create targeted guidelines and messages.

## Introduction

From early in the COVID-19 pandemic, wearing cloth or medical face masks and avoiding crowded public spaces have been important strategies for infection control [1, 2]. Even now, as vaccination and previous infections provide significant protection against the virus and case rates across the U.S. have waned [3], masking remains a valuable strategy for reducing both disease transmission and strain on hospitals [4–6]. 

Considering the important role that mask wearing plays in preventing infections, understanding why people do or do not adhere to public health recommendations like mask wearing is crucial for creating effective recommendations, both in relation to the COVID-19 pandemic and during future health emergencies. The literature on drivers of mask wearing have identified a range of demographic factors associated with increased mask wearing: being female [7–10], living in an urban environment, being older [7, 9] and being Black, Hispanic, or Asian [11, 12]. In addition, beliefs and perceived norms such as: perceived effectiveness of masks [10, 13], perceived risk of infection [10], and seeing other people wear masks [8, 13] have all also been found to increase mask wearing frequency.

Specific drivers of mask wearing behavior have been thoroughly explored in the literature, including work that has utilized the same data used in the current study [8, 11, 13]. However, one overlooked aspect is the survey items that measure self-reported mask wearing. Among most studies in the current literature, participants were asked how often they “wear a mask in public” without considering that people’s behavior may differ while doing different activities in various places (e.g., shopping, visiting friends indoors, visiting friends outdoors, attending a crowded outdoor event).

There is evidence in the health behavior literature supporting the idea that behavior is flexible and context dependent [14–16]. The actions of individuals might be influenced by a range of factors that vary across activities including: whether the activity takes place indoors or outdoors, whether other people are wearing masks and if those people are known to them or are strangers, how risky the person believes the activity to be for contracting an infectious disease, and whether the need for the person to do the activity outweighs other concerns such as getting sick. As a result, even when people intend to behave in a certain way prior to engaging in an activity, they may ultimately choose to act differently based on these external factors.

Prior work has used latent class analyses to identify distinct groups of people by their patterns of engaging in protective health behaviors (such as mask wearing or avoiding activities where infection risk is high) and their beliefs related to COVID-19 [17, 18] but have not considered how aspects of the physical and social environment in which these behaviors take place may play a role. Only two previous studies have been identified that look at mask wearing in different environments, and both found differences in the frequency of mask wearing between activities, with mask wearing being most frequent on public transportation and least common in outdoor settings [19, 20].

In this analysis, we directly compared responses to a question about general mask wearing to responses to several activity-specific mask-wearing questions to assess our first question of whether a general question about masking can meaningfully represent people’s behaviors while engaging in different activities. Second, as a follow-up to our hypothesis that a single question will not be sufficient, as people exhibit a range of different masking behaviors based on what they are doing and where they are doing it, we sought to identify patterns of masking behavior across different activities using latent class analysis and to characterize these patterns in terms of what they reveal about potential motivators and barriers related to mask wearing.

Understanding whether mask wearing is reported with different frequency in different settings by the same individual could significantly change how mask wearing behavior is studied and promoted. Further, if mask wearing is found to vary by setting, understanding which settings drive similar mask wearing behaviors will help public health officials pinpoint contexts and groups that may have challenges that need to be addressed. This is important for the current COVID-19 pandemic as well as future infectious disease outbreaks so that public health recommendations and interventions are shaped by a clear understanding of what matters to people when they are being asked to behave in certain way to protect their and others’ health.

## Method

### Study design, data sources, and variables

The survey datasets were obtained from a commercial market research company through a subscription license [9]. This activity was reviewed by the Centers for Disease Control and Prevention (CDC) and was conducted consistent with applicable federal law and CDC policy [21, 22]. The analytic dataset combined data from four months of national cross-sectional internet panel surveys (November 2020, January 2021, March 2021, May 2021). The surveys were administered bi-monthly to samples of approximately 500 adults (1000 adults in May 2021) not surveyed previously from the continental U.S. Quota sampling and statistical weighting were applied so the dataset reflected the U.S. population by gender, age, census region of the country, race/ethnicity, and education based on Current Population Survey Proportions, a monthly survey of 60,000 households from the U.S. Census Bureau and the Bureau of Labor Statistics [23]. All reported analyses use weighted numbers based on the weights calculated and provided by the market research company with the data [24]. Table 1 provides weighted sample demographics for the analytic sample overall and by survey month. The final analytic sample included 2,508 unique individuals as verified by the market research company. There was no missing data for any of the variables.

### Context of the data collection

Surveys were fielded across the United States, starting in April of 2020, and continued into 2021, with questions concerning people’s experiences of the COVID-19 pandemic. As context is an important factor to understanding how people responded to these surveys, below we have provided a brief review of significant events around the survey collection and encourage readers interested in a more detailed overview to access the CDC Museum’s COVID-19 Timeline [25]. When the first survey that asked about mask wearing in different settings was fielded in November 2020, respondents had been living under a national public health emergency since January 31st, 2020 and the CDC had been recommending people wear face coverings outside their homes in settings where social distancing was not possible since April 3rd, 2020 [25]. Throughout the pandemic, stay at home and face covering orders were enacted at the state and or county levels and their specificity as to whether people were expected to wear masks in all public spaces or only in select circumstances, varied widely [26]. This led to variation based on geography as to both what guidelines people were expected to follow and whether or how these guidelines were communicated and enforced. In November 2020, 29 states had at least limited mask mandates that required people to wear face coverings in some public settings [26].

Additional context to understanding people’s experiences during data collection is vaccine availability, which first became available to certain groups like healthcare workers and the elderly in December of 2020. This means that over the course of the four surveys vaccination rates were increasing. At the time of the final survey in May 2021, 62% of American adults reported having received at least one dose of the vaccine [27]. Average daily case rates, daily death rates, and percent of population fully vaccinated by survey month for respondents’ counties are included in Table 1.

### Sample characteristics

The weighted sample was 52% female with an average age of 47 (*SD* = 18). The majority of respondents were non-Hispanic White (63%), followed by Hispanic (17%) and non-Hispanic Black (12%). Approximately half were married (48%) and employed (55%) and 44% had attended at least some college as their highest level of education.

**Table 1:** Weighted sample demographics by month

Variables	Total	Nov-20	Jan-21	Mar-21	May-21
	*N* = 2,508	*N* = 500	*N* = 501	*N* = 502	*N* = 1,005
**Gender** ^**1**^					
Male	1,211 (48.3%)	241 (48.2%)	242 (48.3%)	242 (48.2%)	485 (48.3%)
Female	1,297 (51.7%)	259 (51.8%)	259 (51.7%)	260 (51.8%)	520 (51.7%)
**Age** ^**2**^	47 (18); 18–93	47 (17); 18–85	47 (18); 18–93	48 (18); 18–90	47 (18); 18–89
**Race/Ethnicity** ^**1**^					
Hispanic	416 (16.6%)	81 (16.2%)	84 (16.8%)	84 (16.7%)	168 (16.7%)
Non-Hispanic White	1,577 (62.9%)	317 (63.4%)	314 (62.7%)	315 (62.7%)	631 (62.8%)
Non-Hispanic Black	299 (11.9%)	59 (11.8%)	60 (12.0%)	60 (12.0%)	120 (11.9%)
Non-Hispanic Native American or Alaska Native	27 (1.1%)	6 (1.2%)	8 (1.5%)	5 (1.0%)	8 (0.8%)
Non-Hispanic Asian	100 (4.0%)	22 (4.5%)	26 (5.3%)	22 (4.5%)	29 (2.9%)
Non-Hispanic Multi-racial or other	88 (3.5%)	14 (2.7%)	9 (1.8%)	16 (3.2%)	50 (4.9%)
**Highest Level of Education** ^**1**^					
High school graduate or less	966 (38.5%)	193 (38.6%)	188 (37.5%)	198 (39.4%)	388 (38.6%)
Some college, 2-year or 4-year degree	1,110 (44.3%)	219 (43.8%)	232 (46.3%)	213 (42.4%)	446 (44.4%)
Some graduate school or attained advanced degree	432 (17.2%)	88 (17.6%)	81 (16.2%)	92 (18.3%)	171 (17.0%)
**Employment Status** ^**1**^					
Employed	1,372 (54.7%)	273 (54.6%)	264 (52.7%)	271 (54.0%)	564 (56.1%)
Not Employed	1,136 (45.3%)	227 (45.4%)	237 (47.3%)	231 (46.0%)	441 (43.9%)
**Type of Community** ^**1**^					
Urban community	889 (35.4%)	168 (33.6%)	171 (34.1%)	183 (36.5%)	366 (36.4%)
Suburban community	1,115 (44.5%)	231 (46.2%)	222 (44.3%)	224 (44.6%)	437 (43.5%)
Rural community	504 (20.1%)	101 (20.2%)	107 (21.4%)	94 (18.7%)	201 (20.0%)
**Census Region**					
Northeast	437 (17.4%)	89 (17.8%)	87 (17.4%)	87 (17.3%)	174 (17.3%)
Midwest	521 (20.8%)	104 (20.8%)	104 (20.8%)	104 (20.7%)	208 (20.7%)
South	951 (37.9%)	188 (37.6%)	190 (37.9%)	191 (38.0%)	382 (38.0%)
West	600 (23.9%)	119 (23.8%)	120 (24.0%)	120 (23.9%)	241 (24.0%)
**Daily count COVID-19 cases in respondents’ counties** ^**3**^	Not Applicable	43,008.79 (75,802.89)	95,806.51 (195,612.90)	119,660.28 (212,897.81)	129,774.07 (233,753.50)
**Daily count COVID-19 related deaths in respondents’ counties** ^**3**^	Not Applicable	1,062.24 (1,824.57)	1,743.66 (3,247.53)	2,306.50 (4,277.88)	2,494.85 (4,736.01)
**Percent population fully vaccinated in respondents’ counties** ^**4**^	Not Applicable	0.00%	0.15%	8.33%	26.94%

^1^n (%)

^2^Mean (SD); Range

^3^Mean (SD), Data sourced from USA facts website [28]

^4^Data sourced from CDC Vaccine tracker [29]

### General mask wearing

The survey contained two questions about mask wearing in general: “In the past week, when you have gone outside of your home for work, grocery shopping, or other activities that involved interacting with other people, how often did you wear a cloth face covering that covered your nose and mouth?” and “…how often did you wear a paper disposable mask, surgical mask, dust mask or other respirator, such as an N95?” Response options were “never, rarely, sometimes, often, always.” Since the target behavior was wearing some kind of face covering and guidance as to what type of mask was effective changed over the course of the pandemic, a single combined measure was created by retaining the more frequent response of the two questions for each respondent (see Table 2 for response frequencies to all mask wearing questions). Respondents who had previously indicated that they had not left their house in the past week were not asked either question.

### Mask wearing by activity

Survey respondents were also asked 12 questions about mask wearing in various activities: “How often did you wear a face covering/mask when doing each of the following activities in the past week?” These activities (listed with their response frequencies in Table 2**)** were selected by social and behavioral science team members who solicited input across COVID-19 emergency response task forces to address questions from the public and to create clearer communications about mask wearing in the midst of the COVID-19 pandemic. Frequencies of the six levels of response (never, rarely, sometimes, often, always, didn’t do this activity) were calculated. In this analysis, the “didn’t do this activity” response is assumed to be a self-protective behavior, as not doing the activity at all confers the highest level of protection. We recognize that this introduces some error to the interpretation. It is not possible to discern the extent to which an individual’s decision not to do a particular activity was motivated by concerns about COVID-19 over other potential reasons, such as the activity not being available due to public health restrictions or an individual having no need to complete the activity in the prior week. However, regardless of the reasoning behind not doing a particular activity the outcome of the behavior is the same: people were more likely to avoid a COVID-19 infection by avoiding interactions with other people in public spaces. Including the “didn’t do this activity” response both preserves sample size for analysis and allows for the inclusion of the greatest level of self-protective behavior.

### Covariates

Several demographic and community level variables were included as covariates in the model. Participants’ gender, age, race/ethnicity, education level, employment status, and community type were included as covariates, as well as the month the survey was administered. Three additional county-level variables were included as covariates: COVID-19 case and death counts (previous day and cumulative from the first local case), and the percentage of eligible people in the county considered fully vaccinated against COVID-19, based on the criteria at the time. County-level variables were matched to respondents based on converting respondents’ zip codes to county level FIPS codes from U.S. Department of Housing and Urban Development-U.S. Postal Service zip code crosswalk file [30].

### Analytic approach

#### Objective #1: Comparing general mask wearing question responses to activity-specific question responses

To determine how consistent participants’ responses were when asked about mask wearing in general compared to during specific activities, a Spearman’s rank order correlation between mask wearing frequency for the general mask-wearing question and frequency for each activity-specific question was calculated. This type of correlation was chosen as the responses to both the general mask wearing and activity specific questions were ordinal and rank ordered ranging from “did not do this activity” (or in the case of the general mask wearing question, did not leave the house in the previous 7 days) to “always” [31]. It was expected that if a general mask wearing question was accurate in capturing people’s mask wearing behaviors, frequency of mask wearing as measured by the general question would be well correlated with frequency of mask wearing during many, if not all, of the 12 activities. We expected that as the consistency of the responses between the two types of questions increased, so would the Spearman’s rank order correlation.

After the correlation was conducted, two sub-samples were created from the main sample to better understand which groups’ behaviors from the general mask wearing questions were most consistent or inconsistent with their responses to the other 12 activity questions: (1) those who chose “always” and (2) those who chose “rarely” or “never” to the general mask wearing question. “Rarely” was included in the second sub-sample due to the small number (*n* = 60) of respondents who chose “never”. Frequencies for mask wearing across the twelve specific activities were then calculated for each sub-sample to identify whether there were environments where people’s responses to the specific questions were inconsistent with the general mask wearing question.

### Objective #2: Identifying patterns of mask wearing behavior in different activities within the respondent sample

To identify patterns among respondents in mask wearing behavior across activities a three-step latent class analysis (LCA) was conducted [32]. LCA allows for the identification of groups of people in a sample who have responded to multiple items in a similar pattern. In this case, an individual’s pattern of mask wearing behaviors across 12 different activities was used to group them with others in the sample who reported behaving in a similar pattern. The survey data was ideal for LCA as we expected people’s behavior while engaged in one activity to be related to their behavior in another activity. For example, someone who “always” wears a mask while shopping at the grocery story may also be more likely to avoid attending large gatherings. LCA has been found to be a more robust method of clustering than other forms of cluster analysis such as k-means clustering [33].

To aid model interpretability, three of the six original response options: “often,” “sometimes,” and “rarely” were collapsed into a single category so that the LCA models included four response categories (“did not do this activity”, “always wore a mask during this activity”, “often, sometimes or rarely wore a mask during this activity” or “never wore a mask during this activity”). This decision was made so that the two most definitive responses “always” and “never” were included in their own categories and the somewhat more ambiguous categories of “often,” “sometimes,” and “rarely” were combined.

First, models of 1 to 9 groups were estimated without covariates and the most optimal number of classes were selected based on model fit statistics, class size, and interpretability. Criteria for model fit statistics involved first examining the Bayesian information criterion (BIC) for each model, which is the best indicator of model fit, and then considering the Akaike information criterion (AIC) and adjusted BIC [34, 35]. For each of these fit indices, lower values indicate better fit though often have a point of diminishing returns for each additional class added. We also confirmed for the selected model that entropy, a measure of separation between classes, was above the recommended 0.80 threshold. Once the optimal number of classes was determined, the covariates listed above were added to the model, fixing the latent class measurement parameters from the model without covariates while accounting for classification error. Latent class modeling was performed in SAS software, version 9.4 using PROC LCA [36] and the SAS %LCA_Covariates_3Step macro [37].

## Results

### Mask wearing behavior in 12 activities

Respondent’s avoidance of activities and their frequency of mask wearing varied across activities: the least frequently engaged in activity was going to the gym (62.9% chose “didn’t do the activity”) and the most frequent activity was shopping for essentials (7.1% “didn’t do the activity”). Similarly, respondents chose “always” wearing a mask when shopping for essentials (57.5%) and least frequently when going to the gym (13.7%). Frequencies for all activities are displayed in Table 2.

**Table 2:** Behavior across activities^1^

Variable	Didn’t do this activity^2^	Always^2^	Often^2^	Sometimes^2^	Rarely^2^	Never^2^
Combined measure of general mask wearing^3^	156 (6.2%)^4^	1342 (53.0%)	576 (23.0%)	308 (12.0%)	66 (2.6%)	60 (2.4%)
Shopping at a store for groceries, prescriptions, or other essential items	178 (7.1%)	1443 (57.5%)	339 (13.5%)	350 (14.0%)	128 (5.1%)	70 (2.8%)
Shopping at a store for non-food items such as clothing or household goods	484 (19.3%)	1168 (46.6%)	236 (9.4%)	340 (13.6%)	201 (8.0%)	79 (3.2%)
Going to a medical or dental appointment	875 (34.9%)	928 (37.0%)	183 (7.3%)	297 (11.9%)	152 (6.1%)	73 (2.9%)
Visiting with family or friends who I do not live with at an indoor setting (e.g., home, restaurant)	892 (35.6%)	400 (15.9%)	261 (10.4%)	349 (13.9%)	275 (11.0%)	331 (13.2%)
Visiting with family or friends who I do not live with at an outdoor setting (e.g., yard, driveway, park)	947 (37.8%)	394 (15.7%)	227 (9.0%)	352 (14.0%)	267 (10.7%)	320 (12.8%)
Eating indoors at a restaurant or bar (e.g., indoor seating area)	1036 (41.3%)	505 (20.1%)	249 (9.9%)	322 (12.9%)	200 (8.0%)	196 (7.8%)
Going to an indoor nail, hair, or personal care salon or spa service provider	1359 (54.2%)	495 (19.7%)	147 (5.9%)	212 (8.5%)	158 (6.3%)	136 (5.4%)
Attending an outdoor gathering with more than 10 people (e.g., sporting event, concert, festival, rally, or protest)	1425 (56.8%)	345 (13.8%)	168 (6.7%)	229 (9.2%)	162 (6.5%)	179 (7.1%)
Attending an indoor gathering with more than 10 people (e.g., sporting event, concert, theatrical or dance performance)	1437 (57.3%)	414 (16.5%)	168 (6.7%)	200 (8.0%)	136 (5.4%)	153 (6.1%)
Attending a faith or religious service indoors	1432 (57.1%)	408 (16.3%)	179 (7.1%)	204 (8.1%)	131 (5.2%)	155 (6.2%)
Using public transportation (e.g., bus or train)	1494 (59.6%)	439 (17.5%)	130 (5.2%)	190 (7.6%)	115 (4.6%)	140 (5.6%)
Going to a gym or indoor recreational facility	1576 (62.9%)	343 (13.7%)	158 (6.3%)	179 (7.1%)	116 (4.6%)	136 (5.4%)

^1^Unless otherwise noted, responses are based on the question “How often did you wear a face covering/mask when doing each of the following activities in the past week?”

^2^n (%)

^3^Combined responses to “In the past week, when you have gone outside of your home for work, grocery shopping, or other activities that involved interacting with other people, how often did you wear a cloth face covering that covered your nose and mouth?” and “…how often did you wear a paper disposable mask, surgical mask, dust mask or other respirator, such as an N95?” choosing the most frequent response

^4^Represents the people who did not leave their house in the previous 7 days and were not asked the general mask wearing question

### Objective #1: Comparing general mask wearing question responses to activity-specific question responses

Responses to the general mask wearing questions ranged from weak to no correlation with frequency of mask wearing in any of the twelve activities based on Spearman’s rank order correlation (*r* =.16–0.33, all correlations were significant at *p* <.05, Table 3). Within the sub-sample of people who chose “always” for the general mask wearing question (*n* = 1,342), frequencies of always wearing a mask for activities that typically require people to leave their home (stores, public transportation, medical appointments, the salon, or the gym) were not fully consistent with the choice of “always” on the general question (ranging from 54 − 75%). For the “rarely/never” group (*n* = 126), a large proportion of respondents reported wearing a mask more frequently than “rarely or never” for each activity. For example, over one third of this group chose “always” wear a mask for visiting stores (39% for essentials and 34% for non-essentials), medical appointments (35%), and salons (30%). Frequencies for both sub-samples are displayed in Table 3.

**Table 3:** Consistency of responses between general mask wearing question and specific settings

	Shopping Essentials	Shopping non-Essentials	Medical	Visiting Indoors	Visiting Outdoors	Restaurants	Going to a Salon	Outdoor Gatherings	Indoor Gatherings	Indoors Religious Services	Public Transportation	Going to a Gym
Correlation	0.33^*^	0.30^*^	0.25^*^	0.16^*^	0.19^*^	0.31^*^	0.27^*^	0.25^*^	0.24^*^	0.27^*^	0.27^*^	0.25^*^
**“Always” Wear a mask on general question (n = 1342)**												
Didn’t do this activity	3.9% (53)	19.0% (256)	36.1% (485)	38.3% (514)	40.1% (538)	45.5% (611)	56.9% (763)	61.3% (822)	61.5% (826)	62.2% (835)	63.2% (848)	66.5% (892)
Always	71.9% (965)	57.3% (769)	43.4% (583)	20.6% (276)	21.2% (284)	25.8% (346)	25.1% (337)	18.1% (243)	20.2% (271)	19.5% (262)	21.2% (284)	18.0% (241)
Often	10.1% (136)	7.1% (95)	5.9% (79)	9.2% (123)	8.2% (109)	9.9% (133)	4.2% (56)	5.3% (71)	5.5% (74)	5.4% (72)	4.3% (58)	4.5% (61)
Sometimes	10.7% (143)	9.6% (129)	8.7% (117)	10.9% (146)	11.2% (150)	8.3% (112)	5.3% (71)	5.8% (77)	4.9% (66)	4.5% (61)	4.3% (57)	4.7% (63)
Rarely	2.4% (33)	5.5% (74)	4.2% (56)	9.2% (124)	8.6% (116)	5.4% (72)	4.6% (61)	4.4% (59)	3.2% (43)	4.0% (54)	3.3% (44)	2.3% (31)
Never	0.9% (12)	1.3% (18)	1.6% (22)	11.8% (158)	10.8% (145)	5.0% (67)	4.0% (54)	5.2% (70)	4.6% (61)	4.3% (57)	3.7% (50)	4.1% (54)
**“Rarely” or “Never” Wear a mask on general question (n = 126)**												
Didn’t do this activity	7.9% (10)	19.0% (24)	46.0% (58)	31.7% (40)	36.5% (46)	38.1% (48)	54.0% (68)	57.1% (72)	54.0% (68)	50.8% (64)	61.9% (78)	56.3% (71)
Always	36.5% (46)	27.8% (35)	19.0% (24)	10.3% (13)	11.1% (14)	8.7% (11)	14.3% (18)	7.1% (9)	10.3% (13)	7.9% (10)	7.1% (9)	9.5% (12)
Often	12.73% (16)	8.4% (11)	7.1% (9)	10.3% (13)	4.8% (6)	6.3% (8)	4.0% (5)	4.8% (6)	4.0% (5)	5.6% (7)	2.4% (3)	7.1% (9)
Sometimes	11.1% (14)	10.3% (13)	9.4% (12)	12.7% (16)	12.7% (16)	13.5% (17)	8.7% (11)	4.8% (6)	7.2% (9)	9.5% (12)	7.9% (10)	4.8% (6)
Rarely	14.3% (18)	19.0% (24)	13.5% (17)	12.7% (16)	12.7% (16)	11.9% (15)	7.1% (9)	10.3% (13)	8.7% (11)	9.5% (12)	11.9% (15)	9.5% (12)
Never	17.5% (22)	14.3% (18)	4.8% (6)	21.4% (27)	23.0% (29)	22.2% (28)	11.9% (15)	15.1% (19)	15.9% (20)	15.9% (20)	8.7% (11)	11.9% (15)

* Correlation is significant at *p* <.05

### Objective #2: Identifying patterns of mask wearing behavior in different activities within the respondent sample

### Model identification

**Fig. 1:**
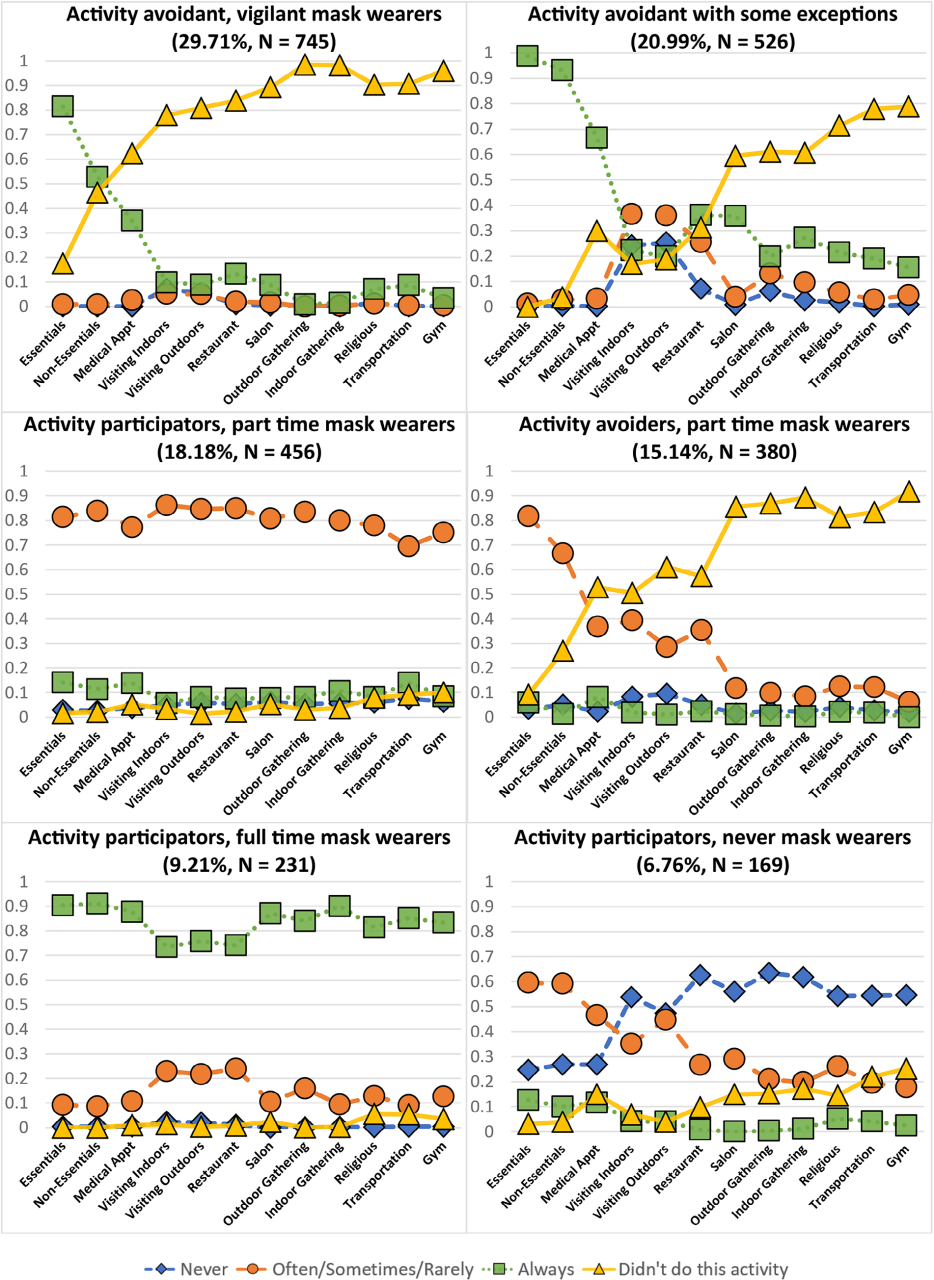
Predicted probabilities of either activity avoidance or mask wearing across activities for the 6-group model

A latent class analysis with all twelve possible activities included as indicator variables and four response categories (“never,” “often/sometimes/rarely,” “always” and “didn’t do this activity”) were estimated with survey population weights applied, starting with a single class, and increasing the number of classes until the BIC and AIC stopped appreciably decreasing. After six classes, the BIC and AIC continued to decrease in only small increments (Table 4, bolded values represent the selected model) and class sizes began to include less than 5% of the sample [35]. To balance both the best fitting model with considerations of interpretability and acceptable class size, six classes were taken as the most appropriate solution for this analysis. Once the best fitting and most interpretable model was determined, covariates were added to this model. Initially, all eleven covariates listed in the Analytic approach section were included. Not all eleven covariates were significant, so non-significant covariates were removed one at a time until only seven significant covariates were included (gender, age, education, employment status, race/ethnicity, month, and percent of county considered fully vaccinated). The predicted probabilities shown in Fig. 1 controlled for the effect of these significant covariates.

**Table 4:** Goodness of fit statistics for LCA models

Classes	Log Likelihood	AIC	BIC	Adjusted BIC	Entropy	df
1	-34401.97	36398.59	36608.36	36493.98	1	16,777,179
2	-28464.08	24596.8	25022.16	24790.22	0.96	16,777,142
3	-25907.4	19557.45	20198.41	19848.91	0.95	16,777,105
4	-24890.87	17598.39	18454.94	17987.88	0.95	16,777,068
5	-24119.31	16129.26	17201.4	16616.79	0.94	16,777,031
**6**	**-23585.28**	**15135.21**	**16422.94**	**15720.77**	**0.92**	**16,776,994**
7	-23283.9	14606.45	16109.78	15290.04	0.9	16,776,957
8	-23048.23	14209.11	15928.03	14990.74	0.91	16,776,920
9	-22859.46	13905.56	15840.07	14785.22	0.91	16,776,883

### Latent classes

Class sizes and probabilities of each response option for each activity are displayed in Fig. 1. For interpretability the six classes were named: activity avoidant, vigilant mask wearers; activity avoidant with some exceptions; activity participators, part time mask wearers; activity avoidant, part time mask wearers; activity participators, full time mask wearers; and activity participators, never mask wearers. The characteristics of membership in each class are described below, in order of class size.

### Activity avoidant, vigilant mask wearers (29.67%)

The largest group in the sample avoided almost all activities except for two of the most necessary (shopping for essentials and medical appointments). For these necessary activities, they were most likely to indicate that they always wore a mask.

### Activity avoidant with some exceptions in terms of social context and in mask wearing (20.80%)

The second largest group was very similar to the highly avoidant group in that they avoided most of the activities and when they engaged in necessary activities like shopping or going to the doctor, they always wore a mask. However, this group’s behavior differed when visiting family and friends (either indoors or outdoors) and eating in restaurants. They seemed to make exceptions for activities where they were more likely to know the people or where wearing a mask was unrealistic (i.e., while eating indoors at a restaurant).

### Activity participators, part time mask wearers (18.18%)

This group was most likely to have chosen often, sometimes, or rarely for all the activities they engaged in.

### Activity avoidant, part time mask wearers (15.27%)

This group was also very similar to the highly avoidant group with a high likelihood that they did not do most of the activities except essentials shopping and medical appointments. However, instead of being likely to always wear a mask while in stores or at the doctor’s office they were more likely to choose often, sometimes, or rarely.

### Activity participators, full time mask wearers (9.21%)

This group was most likely to have chosen always wore a mask for all activities but were unlikely to avoid any activities. Similar to the behavior of the avoidant with exceptions group, there was a slight decrease in the likelihood of the people in this group choosing always wear a mask when visiting family and friends or eating indoors in restaurants.

### Activity participators, never mask wearers (6.77%)

This group was the smallest and the most likely to choose never wear a mask for nearly all of the activities. For shopping and medical appointments, this group was more likely to choose often, sometimes or rarely.

## Discussion

The objectives of this study were to (1) investigate how accurate a general question was in capturing the frequency of people’s mask wearing behavior compared to questions that specify mask wearing during specific activities and (2) identify patterns of mask wearing and avoidance across multiple activities. The results show that there are inconsistencies between how people respond to a general mask wearing question when compared to the frequency of mask wearing during twelve different activities. In addition, the latent class analysis identified six groups of people whose patterns of mask wearing behavior and avoidance of or participation in activities differed distinctly.

The frequency of mask wearing for the general question and for mask wearing during twelve specific activities was weakly correlated. It is possible that in responding to the general question, respondents were choosing a frequency of mask wearing based on their personal support or opposition to mask wearing. There is support in the published literature that social identity can be an important form of self-reporting bias as well as studies confirming that social identity groups (such as political parties) hold strongly differing views about mask wearing [38, 39]. It is also possible that there may be differences in the accuracy of the two types of questions in comparison to people’s actual behavior. The fields of cognitive psychology and survey design have documented the factors involved in a survey respondent’s recall processes when asked to estimate how frequently they engage in a behavior. These findings can provide recommendations to survey designers to help improve recall accuracy among participants [40–42]. One of these suggestions is to provide recall cues that help reduce cognitive load by helping respondents to remember when and where an event happened. Applied to the results of this study, the recall cues provided by the more specific questions that include the places, activities, and people involved may help respondents more accurately remember how frequently they wore a mask compared to the general question, which provides fewer and less specific recall cues and therefore may be more subject to personal opinions towards mask wearing. Looking at the results segmented by those who stated they always wore a mask when leaving the house and those who rarely or never wore a mask suggests that inconsistencies between the general mask wearing question and more specific questions are driven more by the rarely or never group than the always group. The activities in which the rarely or never group were most likely to report always wearing a mask were in spaces like stores, doctor’s offices, and salons. Similarly, in the results of the latent class analysis, the group most likely to indicate they never wear a mask were still likely to report often, sometimes, or rarely wearing masks while shopping or attending doctor’s appointments. Retail or grocery stores and doctor’s offices are spaces that commonly required masks to enter during the COVID-19 pandemic [1]. This finding aligns with prior research indicating health communication campaigns are more effective when they include an enforcement component [43] and more recent work showing that providing periodic in-person reminders about mask policies significantly improved compliance [44].

Two previous studies in the United Kingdom directly measured the frequency of mask wearing across multiple environments [19, 20]. Both studies found that mask wearing was most frequent on public transportation and least common when in outdoor settings [20] or while engaged in leisure activities in indoor settings [19]. The results of both studies suggest that context is an important driver of mask wearing behavior, especially as mask wearing was highest on public transportation, a place where it was required by government policies during the study collection period. Also consistent with our results, an additional study conducted in the United Kingdom found that mask-wearing behavior did not differ when socializing with individuals from other households, regardless of whether the interaction occurred indoors or outdoors [45]. Our study builds upon these findings by studying it with an American sample, expanding the types of activities included and considering how the avoidance of these activities may be a protective action that is related to mask wearing decisions rather than disqualifying the respondents who did not engage in the activity. Our results suggest that surveys of mask wearing behavior should specify where an activity is taking place rather than using a single, global measure. The overall inconsistencies between how people report frequency of mask wearing in general compared to responses for specific environments or activities have implications for how researchers ask about many protective health behaviors, not just mask wearing. It is likely that asking about specific environments in which a behavior takes place may produce more accurate responses by triggering contextual memories compared to less specific memories of recent behavior triggered by a more general question [41, 42].

Our findings from the latent class analysis suggest that the decision of whether to engage in a recommended protective health behavior, like wearing a mask, is a nuanced one and in four of the six groups was related to people’s likelihood to engage in another protective behavior, like avoiding an activity altogether. This is consistent with prior research that showed positive relationships between the likelihood of engaging in one protective health behavior such as mask wearing and other behaviors such as social distancing or hand washing [17, 18, 45, 46]. Evidence about people’s willingness to visit public places during the COVID-19 pandemic indicates that the necessity of the activity may intersect with people’s personal risk assessment and tolerance. Surveys of people’s willingness to engage in various activities found that grocery shopping was the most frequently engaged in activity [9, 47] and a principal component analysis suggested that engagement in this specific activity was fundamentally different for people than other less necessary activities, such as attending large gatherings or indoor dining [18].

The most frequent pattern of behavior in this sample showed that people in the highly avoidant group were most likely to avoid ten of the twelve activities included in the sample and in situations where the activity was necessary, they were likely to wear a mask. Even in the less vigilant groups who made exceptions for activities like visiting friends and family or eating inside restaurants, there are clear interplays between the likelihood of mask wearing and avoiding the activity all together. Consistent with two prior latent class analyses of COVID-19 preventative behaviors [45, 46], the patterns of behavior in three of the groups suggest that people are generally more willing to make exceptions about avoiding or wearing masks during activities that are social in nature where they likely know the people in the environment. It is possible that people may feel there is less risk of infection from a friend or family member compared to situations where they are interacting with strangers [48]. In addition, people’s judgements as to whether they need to engage in a particular activity differ across groups. One group may believe there are greater downsides to not participating in an activity due to financial, opportunity or social cost, despite potential risk for contracting an infectious disease. For example, people have been shown to prioritize social connection over engaging in protective behaviors and prior research has highlighted the negative impacts of social isolation on mental health [49, 50].

The results of this analysis suggest that it is important that public health practitioners remember that people’s decisions about engaging in protective behaviors are not made in a vacuum. They should consider taking a harm reduction approach in messaging so that people are informed about how they can continue to engage in the activities that are most important to them while reducing their risk of infection. These findings suggest scientists need to clearly specify “place” when asking about behaviors like mask wearing, and for public health educators to consider that telling people to “wear a mask” without addressing the “where” may not be effective. Specific recommendations for tailoring messages related to other behaviors are not generalizable from the results of this study but the findings do highlight the importance of context in both messaging and measurement. One approach to ensuring that public health messaging encompasses a wide variety of situations is to increase the role that the community plays in the development of messages [51]. In addition, three previously published works have used the same dataset from the current study [8, 11, 13]. These studies concluded that there are several demographic and behavioral drivers of mask wearing, including belief in the importance of masks and seeing other people wear masks, and that these drivers vary across racial and ethnic groups. It is likely that the groups identified by the LCA also vary in what drives their decision in whether to wear a mask or avoid a specific activity. Future work should consider how differences in these groups could be identified and used to tailor messages that address these drivers.

The current study had several limitations. First, the analysis and interpretation assume that people decided not to engage in specific activities due to concerns about contracting COVID-19. However, it is equally plausible that people did not engage in these activities for a variety of other reasons, including but not limited to: they had no need to engage in the activity in the previous seven days, they never engage in this activity under any circumstances, or the activity was not available due to government restrictions. Future surveys could ask respondents why they avoided a specific activity. Second, the analysis is dependent on self-reported survey data which may be susceptible to social desirability bias where people report more frequent instances of mask wearing. Finally, the data was collected prior to the widespread distribution of the COVID-19 vaccine. It is very likely that these patterns of behavior have changed in the time since May 2021 as people have been able to add an additional layer of protection against COVID-19.

## Conclusion

Although the patterns of behaviors in this analysis may not be generalized to other samples due to historical events and cultural context, the study’s findings emphasize that environmental context is an incredibly important influence on people’s willingness and ability to perform protective health behaviors. As COVID-19 continues to pose a risk and public health continues to focus on preparedness for addressing emerging infectious diseases, the results of this study should help to inform best practices for how to pose questions about behavior. This study adds to the available information that there are identifiable patterns of behavior in mask wearing and avoidance of certain activities, and that people’s decision making around these behaviors are intertwined in important and nuanced ways.

Identifying groups of people who behave similarly across various activities through modeling techniques like latent class analysis can help public health practitioners identify various drivers of behaviors and consider that different groups may have different priorities that need to be considered when messages and other interventions are being developed. This technique is also particularly useful for increasing behaviors that are partially driven by social norms [8, 13]. Information being shared from trusted sources among groups who share collective values and norms related to health are possible points of intervention to improve health behaviors generally, and may be particularly impactful during future public health emergencies [52, 53].

## Data Availability

The data supporting the findings of this study are available from Porter Novelli. The data was licensed for use by CDC and is not publicly available. Individuals seeking access to the data may contact the corresponding author (Ciara Nestor, rta4@cdc.gov) but must also obtain permission from Porter Novelli Public Services (deanne.weber@porternovelli.com).
